# The multiple bonding in heavier group 14 element alkene analogues is stabilized mainly by dispersion force effects†
†Electronic supplementary information (ESI) available: Tables of calculated binding energies for Ge_2_R_4_ and Sn_2_R_4_ (R = CH(SiMe_3_)_2_) with two *syn*, *anti* monomers with optimized and experimental HCECH (E = Ge, Sn) torsion angles; calculated structural data for the Ge_2_R_4_ and Sn_2_R_2_ (R = CH(SiMe_3_)_2_) dimers with the GeR_2_ and SnR_2_ units within the dimers in the *syn*, *syn* configuration; calculated binding energies of the E_2_R_4_ (E = Ge or Sn) dimers with the GeR_2_ and SnR_2_ units in the *syn*, *syn* configuration; calculated structural data for Pb_2_R_4_ with the PbR_2_ units in the *syn*, *anti* configuration; calculated structural and thermodynamic data for the dissociation of [Sn{SiMe^*t*^Bu_2_}_2_]_2_. See DOI: 10.1039/c5sc02707a


**DOI:** 10.1039/c5sc02707a

**Published:** 2015-08-19

**Authors:** Jing-Dong Guo, David J. Liptrot, Shigeru Nagase, Philip P. Power

**Affiliations:** a Fukui Institute for Fundamental Chemistry , Kyoto University , Takano-Nishiraki-cho 34-4 , Sakyo-ku, Kyoto , Japan 606-8103 . Email: nagase@ims.ac.jp; b Department of Chemistry , University of California , One Shields Avenue , Davis , California 95616 , United States . Email: pppower@ucdavis.edu

## Abstract

The structures and bonding in the heavier group 14 element olefin analogues [E{CH(SiMe_3_)_2_}_2_]_2_ and [E{N(SiMe_3_)_2_}_2_]_2_ (E = Ge, Sn, or Pb) and their dissociation into :E{CH(SiMe_3_)_2_}_2_ and :E{N(SiMe_3_)_2_}_2_ monomers were studied computationally using hybrid density functional theory (DFT) at the B3PW91 with basis set superposition error and zero point energy corrections. The structures were reoptimized with the dispersion-corrected B3PW91-D3 method to yield dispersion force effects. The calculations generally reproduced the experimental structural data for the tetraalkyls with a few angular exceptions. For the alkyls, without the dispersion corrections, dissociation energies of –2.3 (Ge), +2.1 (Sn), and –0.6 (Pb) kcal mol^–1^ were calculated, indicating that the dimeric E–E bonded structure is favored only for tin. However, when dispersion force effects are included, much higher dissociation energies of 28.7 (Ge), 26.3 (Sn), and 15.2 (Pb) kcal mol^–1^ were calculated, indicating that all three E–E bonded dimers are favored. Calculated thermodynamic data at 25 °C and 1 atm for the dissociation of the alkyls yield Δ*G* values of 9.4 (Ge), 7.1 (Sn), and –1.7 (Pb) kcal mol^–1^, indicating that the dimers of Ge and Sn, but not Pb, are favored. These results are in harmony with experimental data. The dissociation energies for the putative isoelectronic tetraamido-substituted dimers [E{N(SiMe_3_)_2_}_2_]_2_ without dispersion correction are –7.0 (Ge), –7.4 (Sn), and –4.8 (Pb) kcal mol^–1^, showing that the monomers are favored in all cases. Inclusion of the dispersion correction yields the values 3.6 (Ge), 11.7 (Sn), and 11.8 (Pb) kcal mol^–1^, showing that dimerization is favored but less strongly so than in the alkyls. The calculated thermodynamic data for the amido germanium, tin, and lead dissociation yield Δ*G* values of –12.2, –3.7, and –3.6 kcal mol^–1^ at 25 °C and 1 atm, consistent with the observation of monomeric structures. Overall, these data indicate that, in these sterically-encumbered molecules, dispersion force attraction between the ligands is of greater importance than group 14 element–element bonding, and is mainly responsible for the dimerization of the metallanediyls species to give the dimetallenes. In addition, calculations on the non-dissociating distannene [Sn{SiMe^*t*^Bu_2_}_2_]_2_ show that the attractive dispersion forces are key to its stability.

## Abstract 2

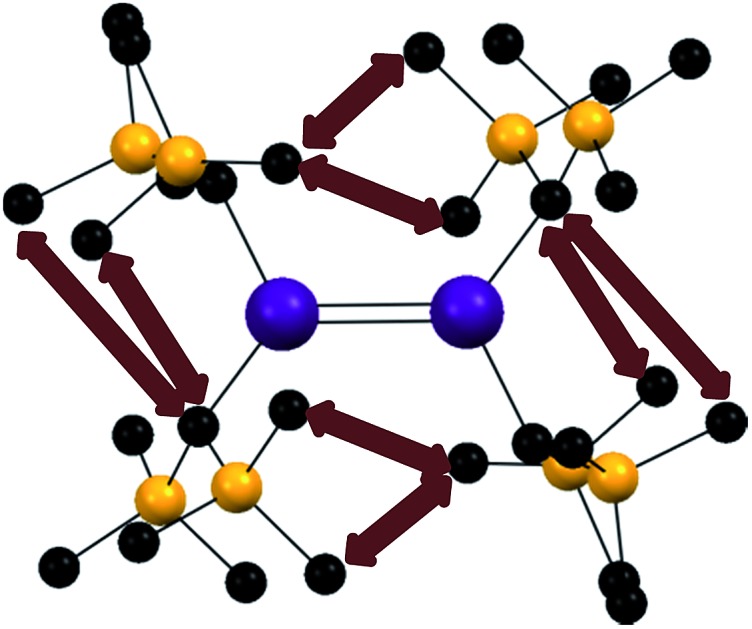
Computations on the heavier group 14 dimetallenes [E{CH(SiMe_3_)_2_}_2_]_2_ and [E{N(SiMe_3_)_2_}_2_]_2_ (E = Ge, Sn, or Pb) and their respective monomers indicated that empirically observed dimerization is principally driven by attractive dispersion forces.

## Introduction

**Fig. 1:**
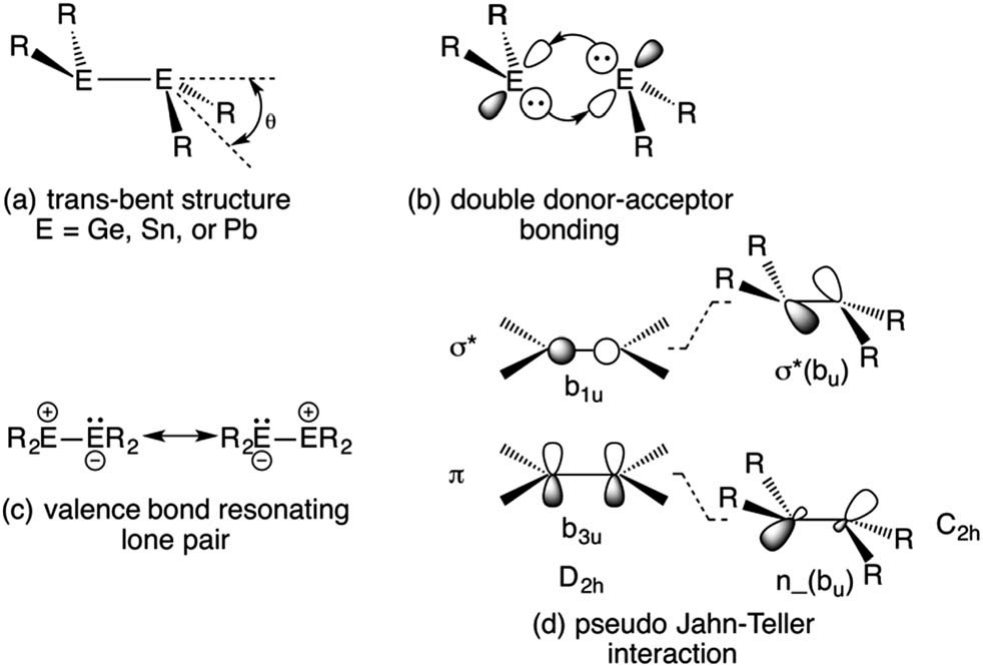
*Trans*-pyramidalized geometry (a) and bonding models (b–d) for heavier group 14 element olefin analogues.

The synthesis of the lower valent group 14 element dialkyls :E{CH(SiMe_3_)_2_}_2_ (E = Sn and Pb) in 1973 1 and the corresponding isoelectronic amido derivatives: E{N(SiMe_3_)_2_}_2_ (E = Ge, Sn, and Pb) in 1974 2,3 were landmark events in the evolution of modern main group chemistry. The alkyl derivatives in particular were to exert a great influence on the perception of bonding between heavier main group elements because of their unprecedented structures and chemical behavior. Both the amides and alkyls were shown to exist as monomers in benzene solution^1^,^2^ and in the vapor phase.^4^–^6^ But whereas the amides remained monomeric as solids,^5^,^7^ the alkyl derivatives displayed unusual E–E bonded dimeric structures in the solid state.^8^–^11^ Although they are heavier congeners of substituted ethylenes, their group 14 atoms were found to have non-planar coordination, and the structures displayed a centrosymmetric *trans*-pyramidalized (or folded) configuration (Fig. 1a), as well as E–E bond lengths that did not display the extent of shortening expected for double bond formation.^8^–^11^ The original interpretation of the (at the time) peculiar E–E bonding was based on either a double donor–acceptor bond (Fig. 1b) or a valence bond resonance structure (Fig. 1c). The bonding can be viewed also in terms of a pseudo (also called 2^nd^-order) Jahn–Teller mixing^12^,^13^ of the π-bonding and σ* levels which occurs upon a *trans*-bending distortion (*i.e.* a vibration) of the E–E frameworks. The mixing of a bonding and antibonding orbital generates a molecular orbital with lone-pair character, and hence a pyramidalized coordination for E. This interaction takes place more readily in the heavier main group element derivatives because the bonds become increasingly long (weaker) as a result of larger Pauli repulsions between core electrons as the group is descended. Consequently, the energy difference between the σ* and π orbitals also decreases, and hence the extent of their interaction increases. The *trans*-folding, which weakens the E–E bond, is often sufficient to cause dissociation of the double bond of the heaviest tin and lead species to two monomeric metallanediyls. However, dissociation is less common in their germanium analogues and for the iconic disilenes, first reported in 1981,^14^ dissociation is rare and the silicon coordination is generally close to planar.^15^–^17^ In addition to the aforementioned bonding models, there is the broadly-applicable CGMT approach,^18^ which focuses on the energetics of double bond formation. It is based on the fact that the double bonded species are formed from two constituent carbene-like metallanediyl units. This allows the bond energies and molecular geometry to be rationalized in terms of the singlet–triplet excitation energies of the metallanediyl monomers.^19^

The investigation and rationalization of bonding in these and related multiple-bonded compounds has been a topic of broad interest^15^–^17^ that continues to the present day.^20^ These compounds, and others in neighboring groups,^21^ marked a departure from the widely-assumed notion that heavier main group elements did not form double or triple bonds to each other.^22^ The key development that enabled their stabilization was the use of sterically large hydrocarbon substituents^23^ that blocked their decomposition *via* association or elimination reactions. In the numerous reviews and discussions of heavier main group multiple bonds, the focus has been almost exclusively on details of the interaction between the two heavier elements, *i.e.* the orbitals likely to be involved in the multiple bonds, their relative energies, their likely bonding character, and the extent of the overlap. However, recent work, mainly on carbon compounds that carry bulky substituents,^24^,^25^ has shown that attractive dispersion interactions between the C–H moieties of the substituents also play a large role in the stabilization of sterically crowded molecules. This attraction is exemplified by the stabilization of the substituted ethane {C(C_6_H_3_-3-,5-Bu^*t*^_2_)_3_}_2_, which has a long central single C–C bond of 1.67 Å and is stabilized *via* dispersion force interactions between the Bu^*t*^ groups, whereas the corresponding C–C bonded, less hindered, unsubstituted species (CPh_3_)_2_ (*i.e.* the C–C bonded dimer of the Gomberg radical) is unknown.^26^

Despite these developments, the realization of importance of the dispersion interactions between the C–H moieties of substituent ligands to the stability of inorganic and organometallic compounds is not widespread. However, several reports have shown that such forces are very important for the bonding and structure of a variety of species.^24^–^35^ We showed recently that attractive dispersion forces were of key importance in the association of sterically crowded phosphinyl and arsinyl radicals to the corresponding diphosphanes and diarsanes.^35^ We now describe the results of density functional theory calculations with and without dispersion force contributions for the monomeric dialkyls and diamides E{CH(SiMe_3_)_2_}_2_ and E{N(SiMe_3_)_2_}_2_ (E = Ge, Sn, or Pb) as well as the corresponding E–E bonded dimers. The calculations without the inclusion of dispersion force effects revealed that the E–E bonding energies are low for the tetraalkyls, and insufficient to stabilize their dimeric structures under ambient conditions. In contrast, the inclusion of dispersion force resulted in large increases in the binding energies. Application of the same protocols to the amido compounds also afforded lower binding energies that are insufficient to sustain the dimeric structures under ambient conditions.

## Experimental section

### Computational methods

All calculations were carried out using the Gaussian 09 program.^36^ Geometry optimization was performed with hybrid density functional theory (DFT) at the B3PW91 (ref. 37) level by using the 6-311+G(2d) basis set for Ge, the [4333111/433111/43] basis set augmented by two d polarization functions (d exponents 0.253 and 0.078) for Sn,^38^ the SDD basis set and its effective core potential for Pb,^39^ and the 6-31G(d,p)^38^,^39^ basis set for other atoms. In order to estimate the dispersion effects, geometries were reoptimized with the dispersion-corrected B3PW91-D3 method.^40^ Geometry optimization was also performed at the B97-D3 level.^40^,^41^ Single-point MP2 (second-order Møller–Plesset perturbation theory)^42^ energy calculations were performed on B3PW91-D3 optimized geometries. The basis set superposition errors (BSSE) were estimated with the counterpoise method.^43^ Enthalpy (*H*), entropy (*S*), and free energies (*G*) as well as zero point energies (ZPE) were estimated using calculated harmonic vibrational frequencies and BSSE corrected energies.

## Results and discussion

### Computational data for the alkyl monomers and dimers, E{CH(SiMe_3_)_2_}_2_ and [E{CH(SiMe_3_)_2_}_2_]_2_ (E = Ge, Sn, or Pb)

**Fig. 2:**
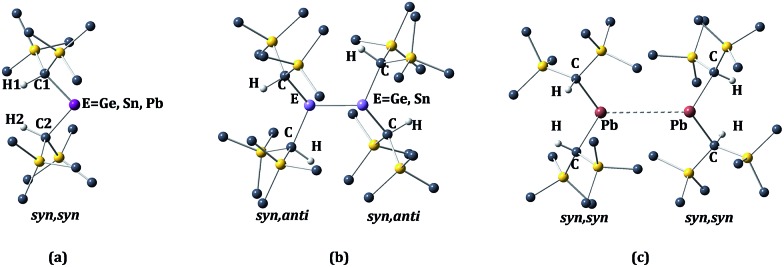
*Syn*, *syn* (a) and *syn*, *anti* (b) configurations for the monomer E{CH(SiMe_3_)_2_}_2_ and dimer [E{CH(SiMe_3_)_2_}_2_]_2_ (E = Ge, Sn, or Pb).

The calculated structural parameters at various levels of optimization for the alkyl-substituted germanium, tin, and lead monomers and dimers are presented in Table 1 and 2 respectively. The E{CH(SiMe_3_)_2_}_2_ (E = Ge or Sn) monomers have the *syn*, *syn* orientation of the –CH(SiMe_3_)_2_ alkyl groups in the gas phase, as shown in Fig. 2a. At present, no gas phase electron diffraction data are available for the Pb{CH(SiMe_3_)_2_}_2_ monomer. However, in Table 1 we have given the experimental parameters for Pb{CH(SiMe_3_)_2_}_2_, derived from a *syn*, *syn* Pb{CH(SiMe_3_)_2_}_2_ unit within the [Pb{CH(SiMe_3_)_2_}_2_]_2_ (ref. 11) ‘dimer’. In the X-ray structures of the germanium and tin crystalline dimers the E{CH(SiMe_3_)_2_}_2_ (E = Ge or Sn)^8^–^10^ units have a *syn*, *anti* conformation, as shown in Fig. 2b. In contrast, the Pb{CH(SiMe_3_)_2_}_2_ units within the lead ‘dimer’ have the aforementioned *syn*, *syn* configuration, as shown in Fig. 2c, *i.e.* the same configuration as those experimentally observed for the germanium and tin dialkyl monomers in the gas phase.^10^ The calculated bond lengths and angles are generally close to those observed experimentally. This statement is particularly true for the X-ray crystal structures of the germanium and tin dimers, where the experimental structural parameters and E–E bond lengths (Ge–Ge = 2.347(2) Å^9^; Sn–Sn = 2.768(1) Å^10^) are reproduced with good accuracy. Exceptions to this generalization involve the calculated torsion angles between the methane C–H bonds and the central EC_2_ (E = Ge or Sn) plane in the germanium and tin monomers, and the C–Ge–C angle in the germanium monomer, which is somewhat high. For the torsion angle in the germanium and tin monomers, the discrepancy is almost 20° in the germanium and 6–8° in the tin monomer. Unfortunately, in the monomeric structures which are measured from GED (gas electron diffraction) data, no standard deviations were given. However, standard deviations of 2° were listed for the EC_2_ angle, and it is reasonable to suppose the standard deviation for the torsion angle for the methane C–H bonds would be larger than this value. We replicated the optimization with the HCECH torsion angles fixed at the experimental values of 2° (E = Ge) and 15° (E = Sn). As shown in Table S1,† this increased the binding energy by 10.7 kcal mol^–1^ for Ge, but only 2.5 kcal mol^–1^ for tin. Thus, the HCECH torsion angle has significantly larger effect in the germanium dimer, consistent with its more sterically-congested structure. It is worthwhile to recall that the calculations on the dimers are for the molecules in isolation (*i.e.* in the gas phase), and take no account of the effects of neighboring molecules (*i.e.* packing forces), which can also be expected to exert some effect on their structure.

**Table 1:** Calculated and experimental structural data for E{CH(SiMe_3_)_2_}_2_ (E = Ge, Sn, or Pb) monomers in *syn*, *syn* configuration

	Ge{CH(SiMe_3_)_2_}_2_				Sn{CH(SiMe_3_)_2_}_2_				Pb{CH(SiMe_3_)_2_}_2_			
	B3PW91	B3PW91-D3	B97-D3	Exp^*a*^	B3PW91	B3PW91-D3	B97-D3	Exp^*a*^	B3PW91	B3PW91-D3	B97-D3	Exp^*b*^
E–C (Å)	2.014	2.003	2.039	2.038(15)	2.232	2.217	2.252	2.22(2)	2.368	2.343	2.365	2.318(5)
Si–C1 (Å)	1.903 (avg.)	1.887	1.904 (avg.)	1.896(3)	1.893 (avg.)	1.876 (avg.)	1.892 (avg.)	1.897(3)	1.885 (avg.)	1.868 (avg.)	1.885 (avg.)	1.862 (avg.)
C1–E–C2 (°)	101.4	98.2	98.0	107(2)	98.4	94.4	94.2	97(2)	96.8	92.2	92.0	93.4(2)
E–C1–Si (°)	109.7 (avg.)	109.0	110.0 (avg.)	110.6(6)	109.7 (avg.)	108.4 (avg.)	109.7 (avg.)	109.7(7)	109.4	107.9 (avg.)	109.6 (avg.)	109.5 (avg.)
Si–C1–Si (°)	116.1	116.1	116.4	113.0(5)	117.4	117.7	117.8	114.0(3)	118.4	118.6	118.4	109.8
C1–E–C2–H2 (°)	21.3	21.8	21.4	2	21.7	23.5	22.8	15	22.6	23.6	22.9	20

^*a*^Gas Electron Diffraction (GED); ref. 6.

^*b*^Data are from a monomeric Pb{CH(SiMe_3_)_2_}_2_ within the crystal structure of its weakly associated dimer; ref. 11.

**Table 2:** Calculated and experimental structural parameters for the dimetallenes [E{CH(SiMe_3_)_2_}_2_]_2_ (E = Ge, Sn, or Pb), in which the E{CH(SiMe_3_)_2_} units have the *syn*, *anti* (E = Ge or Sn) or *syn*, *syn* (Pb) configuration

[Ge{CH(SiMe_3_)_2_}_2_]_2_				
	B3PW91	B3PW91-D3	B97-D3	X-ray^*a*^
E–E (Å)	2.373	2.315	2.376	2.347(2)
E–C (Å)	2.022 (avg.)	2.001	2.042 (avg.)	2.01(3)
C–E–C (°)	109.9	111.8	111.0	112.5(3)
C–E–E (°)	114.3	113.8	112.6	113.7(3)
	122.5	120.5	119.8	122.3(2)
C–E–E–C (°)	–43.0	–43.1	–46.6	–39.5(3)
	180.0	180.0	180.0	180.0(3)
*Trans*-bending angle (°)	34.3	35.7	38.8	32

[Sn{CH(SiMe_3_)_2_}_2_]_2_				
	B3PW91	B3PW91-D3	B97-D3	X-ray^*b*^
E–E (Å)	2.777	2.694	2.774	2.768(1)
E–C (Å)	2.230 (avg.)	2.198	2.255 (avg.)	2.216 (avg.)
C–E–C (°)	109.8	111.2	109.8	109.2(2)
C–E–E (°)	113.5	113.4	112.2	112.0(1)
	120.8	118.2	117.6	119.4(1)
C–E–E–C (°)	–46.4	–47.2	–51.2	–50.5(1)
	180.0	180.0	180.0	180.0(1)
*Trans*-bending angle (°)	37.6	39.7	42.9	41

[Pb{CH(SiMe_3_)_2_}_2_]_2_				
	B3PW91	B3PW91-D3	B97-D3	X-ray^*c*^
E–E (Å)	6.673	3.241	3.256	4.129(1)
E–C (Å)	2.368 (avg.)	2.357 (avg.)	2.383 (avg.)	2.313(5)
				2.323(5)
C–E–C (°)	96.8 (avg.)	91.3 (avg.)	91.8 (avg.)	93.4(2)
C–E–E (°)	122.7 (avg.)	116.9 (avg.)	118.0 (avg.)	
C–E–E–C (°)	–54.6 (avg.)	–73.8 (avg.)	–71.5 (avg.)	–55.70
	171.3 (avg.)	179.2 (avg.)	179.3 (avg.)	180.0(1)
*Trans*-bending angle (°)	35.6	49.9	47.8	34

^*a*^ Ref. 9.

^*b*^ Ref. 10.

^*c*^ Ref. 11.

The calculated Pb–Pb separation and C–Pb–C angle in the dimeric lead structure differ considerably from the experimentally measured values. The experimental Pb–Pb distance of 4.129(1) Å in the crystal structure of the [Pb{CH(SiMe_3_)_2_}_2_ dimer^11^ is far longer than the sum of single bond covalent radii of 2.88 Å expected for a Pb–Pb single bond.^44^ Furthermore, the calculated Pb–Pb distances using the B3PW91-D3 method is 3.241 Å and, by the B97-D3 method, it is 3.256 Å. These distances are over 1 Å shorter than the experimentally measured value, and suggest a very weak interaction between the lead atoms of the Pb{CH(SiMe_3_)_2_}_2_ units in the crystal structure. Nevertheless, long interactions of this type between the heavier main group elements can be significant.^45^ In spite of the observed deviation in Pb–Pb separation, the experimental and calculated C–Pb–C angles of the lead dimer are in good agreement with each other. If it is assumed that the Pb{CH(SiMe_3_)_2_}_2_ units within the lead dimer are *syn*, *syn* monomers, that is to say the long Pb–Pb interaction is ignored, the structural parameters given for Pb{CH(SiMe_3_)_2_}_2_ in Table 1 are obtained. It can be seen that the calculated C–Pb–C angles of 96.6° (B3PW91), 92.2° (B3PW91-D3), or 92.0° (B97-D3) are also close to the experimentally-measured value of 93.4(2)°. Moreover, this angle also resembles those calculated (range 98.4–94.2°) for the C–Sn–C angle in the Sn{CH(SiMe_3_)_2_}_2_ monomer (*cf.* C–Sn–C = 97(2)° by GED). In effect, the data suggest that the Pb{CH(SiMe_3_)_2_}_2_ units within the lead dimer are behaving essentially as weakly interacting plumbylene monomers, rather than as a Pb–Pb multiple bonded diplumbene.

It was noted above that the experimentally-determined orientation of the –CH(SiMe_3_)_2_ groups of the E{CH(SiMe_3_)_2_}_2_ units within the germanium and tin dimers are *syn*, *anti* and the computational data in Table 2 and 3 have been made on the basis of this orientation. However, it is possible to perform similar computations with the assumption of *syn*, *syn* orientation in the E{CH(SiMe_3_)_2_}_2_ units. The results of these calculations are given in Tables S2 and S3.† The changed orientation results in longer E–E distances and smaller binding energies of 18.9 (Ge) and 20.0 (Sn) kcal mol^–1^ at the B3PW91-D3 level with BSSE and ZPE corrections, which can be compared to the values of 28.7 (Ge) and 26.3 (Sn) kcal mol^–1^ in Table 3. In effect, the changed orientation of the substituents results in binding energies that are *ca.* 10 kcal mol^–1^ lower than the *syn*, *anti* configuration values. These energy differences are somewhat smaller than the 16.4 kcal mol^–1^ calculated for the corresponding changes in the energy of the [P{CH(SiMe_3_)_2_}_2_]_2_ → 2P{CH(SiMe_3_)_2_}_2_ process upon relaxation of the configurations from *syn*, *anti* to *syn*, *syn*.^35^ The difference in values can be rationalized, at least in part, in terms of the reduced inter E{CH(SiMe_3_)_2_}_2_ unit steric crowding because of the larger sizes of germanium and tin in comparison to that of phosphorus.

**Table 3:** Thermodynamic data (kcal mol^–1^) for the dissociation of the dimetallenes [E{CH(SiMe_3_)_2_}_2_]_2_ into two metallanediyl *syn*, *syn* monomers, E{CH(SiMe_3_)_2_}_2_

[E{CH(SiMe_3_)_2_}_2_]_2_ → 2E{CH(SiMe_3_)_2_}_2_									
	E = Ge			E = Sn			E = Pb		
	B3PW91	B3PW91-D3	B97-D3	B3PW91	B3PW91-D3	B97-D3	B3PW91	B3PW91-D3	B97-D3
Δ*E*^*a*^	5.6	40.2 (41.2)	33.9	12.0	38.5 (41.5)	33.3	0.1	22.1 (10.3)	20.2
Δ*E*^*b*^	–2.3	28.7		2.1	26.3		–0.6	15.2	
Δ*H*	–2.3^*c*^, –2.7^*d*^	30.1^*c*^, 29.9^*d*^		2.2^*c*^, 2.0^*e*^	27.0^*c*^, 26.9^*e*^		–1.5^*c*^	15.2^*c*^	
–*T*Δ*S*	–15.5^*c*^, –29.4^*d*^	–20.7^*c*^, –37.1^*d*^		–17.0^*c*^, –29.1^*e*^	–19.9^*c*^, –33.0^*e*^		–8.4^*c*^	–16.9^*c*^	
Δ*G*	–17.8^*c*^, –32.1^*d*^	9.4^*c*^, –7.2^*d*^		–14.8^*c*^, –27.1^*e*^	7.1^*c*^, –6.1^*e*^		–9.9^*c*^	–1.7^*c*^	

^*a*^Dissociation energy (kcal mol^–1^). MP2 values are in parentheses.

^*b*^With ZPE and BSSE corrections.

^*c*^At 25 °C (298 K) and 1 atm.

^*d*^At 155 °C (428 K) and 0.1 Torr.

^*e*^At 120 °C (393 K) and 0.1 Torr.

It is also informative to consider the effect of altering the orientation of the CH(SiMe_3_)_2_ groups of the [Pb{CH(SiMe_3_)_2_}]_2_ dimer to the *syn*, *anti* orientation observed for the lighter congeners; the data in Table S4† reflect calculations in this orientation. The resultant Pb–Pb distance is consistently shorter than in both the experimental and computational results in the *syn*, *syn* orientation; the dispersion corrected distance of 2.956 Å (B3PW91-D3) approaches the sum of the covalent radii indicating a significant degree of Pb–Pb bonding. Also noteworthy is the effect upon the C–Pb–C angle which is calculated to widen significantly (91.3 *versus* 106.6° at the B3PW91-D3 level); likely a consequence of steric clash between the SiMe_3_ groups of the ligand. Such an angle, however, would also optimize Pb–Pb bonding by increasing the p-character of the Pb lone pair. That the Pb{CH(SiMe_3_)_2_} dimer does not adopt such a conformation must suggest the nature of the Pb{CH(SiMe_3_)_2_}_2_ dimer is that of two weakly interacting plumbylene monomers wherein significant s-character of the lone pair enforced by the narrow C–Pb–C angles (93.4(2)°) lead to “closed-shell” interactions between the Pb atoms.

### Calculated thermodynamic data for the dissociation of the [E{CH(SiMe_3_)_2_}_2_]_2_ dimers

The data for the dissociation of the dimeric [E{CH(SiMe_3_)_2_}_2_]_2_ to two E{CH(SiMe_3_)_2_}_2_ monomers are summarized in Table 3. Without inclusion of dispersion effects, it can be seen that the binding energies are –2.3, 2.1, and 0.6 kcal mol^–1^ for the germanium, tin, and lead dimers with BSSE and ZPE corrections. The low values result from steric repulsion between the –CH(SiMe_3_)_2_ groups. However, inclusion of dispersion effects using the B3PW91-D3 approach dramatically increases the binding energies to 28.7, 26.3, and 15.2 kcal mol^–1^, respectively. With the B97-D3 method, the calculated energies are 33.9, 33.3, and 20.2 kcal mol^–1^. Single-point MP2-based calculations afford energies of 41.2, 41.5, and 10.3 kcal mol^–1^. Taken together, the calculated energies strongly suggest that the major portion of the binding energy for the germanium, tin, and lead dimetallenes are a result of attractive dispersion forces between the –CH(SiMe_3_)_2_ ligands. Calculation of the free energy changes (Δ*G*) at 25 °C and 1 atm for the dissociation of the dimers, without dispersion force correction, affords values of –17.8, –14.8, and –9.9 kcal mol^–1^ for the germanium, tin, and lead derivatives respectively. In other words, dissociation is favored in all cases. Application of the dispersion-corrected B3PW91-D3 method changes these Δ*G* values to +9.4, +7.1, and –1.7 kcal mol^–1^. Thus, the dimeric structures become favored for the germanium and tin species, but remains slightly disfavored for lead. These findings are in accord with the relatively long element–element distances experimentally observed for the germanium and tin dimers, and the very weak interaction in the case of the lead species. The Δ*G* energies calculated for the dissociation of germanium and tin alkyls at the temperatures and pressures at which the GED data sets were collected, *i.e.* 428 K and 0.1 Torr for the germanium dialkyl and 393 K and 0.1 Torr for the tin dialkyl, were –7.2 and –6.1 kcal mol^–1^. These negative values are consistent with the monomeric structures observed in the vapor phase.

There has been only one report of an experimental determination of energies associated with the monomer–dimer equilibrium of the E_2_R_4_ (E = Ge, Sn, or Pb; R = CH(SiMe_3_)_2_) series.^46^ This was accomplished by variable-temperature NMR spectroscopy of [Sn{CH(SiMe_3_)_2_}_2_]_2_ and the behavior of the ^13^C shifts for the methine carbons in the monomers and dimers. This allowed calculation of Δ*H* = 12.8 kcal mol^–1^ and Δ*S* = 33 cal K^–1^ mol^–1^ for the dissociation. These experimental values differ considerably from those calculated (*cf.* Δ*H* = 27.0 kcal mol^–1^ and Δ*S* = 66.7 cal K^–1^ mol^–1^). At present, the reason for the discrepancy between the experimental and calculated values is unclear, and more data will be required to establish the expected values for similarly-substituted monomer–dimer equilibria. For example, significantly higher Δ*S* values of 75 and 66 cal mol^–1^ K^–1^ have been determined for the dissociation of the [M{N(SiMe_3_)_2_}_2_]_2_ (M = Fe or Co), which carry –N(SiMe_3_)_2_ substituents that are isoelectronic to CH(SiMe_3_)_2_.^47^,^48^ The dissociation of the digermene [Ge(C_6_H_2_-2,4,6-Me_3_){C_6_H_2_-2,4,6-(CH(SiMe_3_)_2_)_3_}]_2_ was studied by electronic spectroscopy which revealed a Δ*H* value of 14.7(2) kcal mol^–1^ and a Δ*S* value of 42.4 cal mol^–1^ K^–1^.^49^

A rationalization for the observed congeneric variation can thus be constructed wherein the interplay between the effects of dispersion interactions, steric congestion and metal–metal bond strength define the observed structures of the [E{CH(SiMe_3_)_2_}_2_]_2_ (E = Ge, Sn, Pb) dimers. The *syn*, *anti* conformation adopted by germanium and tin yields an increased interligand steric congestion,^33^ and thus other factors must stabilize the adoption of this orientation in the dimers. In contrast, such stabilization must be insufficient in the case of the lead congener which results in the adoption of a less crowded *syn*, *syn* orientation and consequent attenuation of metal–metal bonding. This conclusion is supported by the fact that the seven-membered ring dialkyl lead(ii) species <svg xmlns="http://www.w3.org/2000/svg" version="1.0" width="16.000000pt" height="16.000000pt" viewBox="0 0 16.000000 16.000000" preserveAspectRatio="xMidYMid meet"><metadata> Created by potrace 1.16, written by Peter Selinger 2001-2019 </metadata><g transform="translate(1.000000,15.000000) scale(0.005147,-0.005147)" fill="currentColor" stroke="none"><path d="M0 1960 l0 -760 80 0 80 0 0 680 0 680 1280 0 1280 0 0 80 0 80 -1360 0 -1360 0 0 -760z"/></g></svg> PbC(SiMe_3_)_2_SiMe_2_CH_2_CH_2_SiMe_2_C <svg xmlns="http://www.w3.org/2000/svg" version="1.0" width="16.000000pt" height="16.000000pt" viewBox="0 0 16.000000 16.000000" preserveAspectRatio="xMidYMid meet"><metadata> Created by potrace 1.16, written by Peter Selinger 2001-2019 </metadata><g transform="translate(1.000000,15.000000) scale(0.005147,-0.005147)" fill="currentColor" stroke="none"><path d="M0 2640 l0 -80 1280 0 1280 0 0 -680 0 -680 80 0 80 0 0 760 0 760 -1360 0 -1360 0 0 -80z"/></g></svg> (SiMe_3_)_2_,^50^ which has a very similar substitution pattern to that in [Pb{CH(SiMe_3_)_2_}_2_]_2_ and a C–Pb–C angle of 117.1(2)°, has no Pb–Pb contact shorter than 8.911 Å. Furthermore, the Sn(ii) dialkyl <svg xmlns="http://www.w3.org/2000/svg" version="1.0" width="16.000000pt" height="16.000000pt" viewBox="0 0 16.000000 16.000000" preserveAspectRatio="xMidYMid meet"><metadata> Created by potrace 1.16, written by Peter Selinger 2001-2019 </metadata><g transform="translate(1.000000,15.000000) scale(0.005147,-0.005147)" fill="currentColor" stroke="none"><path d="M0 1960 l0 -760 80 0 80 0 0 680 0 680 1280 0 1280 0 0 80 0 80 -1360 0 -1360 0 0 -760z"/></g></svg> SnC(SiMe_3_)_2_(CH_2_)_2_C <svg xmlns="http://www.w3.org/2000/svg" version="1.0" width="16.000000pt" height="16.000000pt" viewBox="0 0 16.000000 16.000000" preserveAspectRatio="xMidYMid meet"><metadata> Created by potrace 1.16, written by Peter Selinger 2001-2019 </metadata><g transform="translate(1.000000,15.000000) scale(0.005147,-0.005147)" fill="currentColor" stroke="none"><path d="M0 2640 l0 -80 1280 0 1280 0 0 -680 0 -680 80 0 80 0 0 760 0 760 -1360 0 -1360 0 0 -80z"/></g></svg> (SiMe_3_)_2_,^51^ featuring the tin atom in a five-membered ring structure and a C–Sn–C angle of 86.7(2)°, is also monomeric. Here the C(SiMe_3_)_2_ moieties have a *syn*, *syn*-like conformation analogous to the vapor phase structure of Sn{CH(SiMe_3_)_2_}_2_ and the lead congener in all cases. These data suggest that a *syn*, *syn* orientation precludes strong M–M contact and thus any dimerisation must emerge principally from dispersion interactions.

In the case of the germanium and tin congeners, two factors clearly stabilize the sterically unfavorable adoption of a *syn*, *anti* orientation; the strength of dispersion forces and the degree of metal–metal bonding. The dispersion interactions in these dimers is evidently optimized between different ER_2_ fragments favoring dimerisation, as evidenced by the increase in C–E–C angle when dispersion corrected (Ge = 109.9° (B3PW91), 111.8° (B3PW91-D3); Sn = 109.8° (B3PW91), 111.2° (B3PW91-D3)). These data reinforce that dispersion effects are the principal driving force for dimerisation in these species and act as a scaffold for metal–metal bonding.

In contrast, the application of a dispersion correction to the lead structure yields, in the case of the experimentally consistent *syn*, *syn* orientation a contraction of the C–M–C angle (96.8° (B3PW91), 91.3° (B3PW91-D3)) or in the case of the putative *syn*, *anti* orientation a negligible change (106.3° (B3PW91), 106.6° (B3PW91-D3)). These data are a likely consequence of the increase in E–C bond length down group 14, precluding dimer-favoring dispersion interactions in the Pb case, and instead favoring intramonomer interactions which cannot stabilize the steric clash introduced by a *syn*, *anti* orientation of the ligands. Furthermore, this narrowing of the C–Pb–C angle increases the s-character of the Pb centered lone pair, disfavoring metal–metal bonding further indicating that any Pb–Pb interactions are closed-shell in nature.

### The amido monomers E{N(SiMe_3_)_2_}_2_ (E = Ge, Sn, or Pb)

The calculated and experimental structural parameters for the three monomeric amido derivatives E{N(SiMe_3_)_2_}_2_ (E = Ge, Sn, or Pb) are provided in Table 4. Diffraction data for both the gas (GED) and crystalline phases (X-ray), which show that each has a monomeric structure, are listed. The experimental data for the gas and crystalline phases differ considerably in their N–E–N angles and the dihedral angles between the EN_2_ and NSi_2_ coordination planes. It can also be seen that the N–E–N angle is much more sensitive to the identity of the central atom (E) in the vapor phase than in the solid state. Furthermore, the N–E–N angle is significantly narrower for the tin and lead compounds in the vapor phase than in the solid state. Such differences have been attributed to the changed ligand orientation in the gas phase, where the NSi_2_ planes are essentially perpendicular to the EN_2_ plane.^5^,^7^ This may minimize steric repulsion because of the parallel orientation of the NSi_2_ planes. However, it would give inefficient packing in the solid state. As a result, the –NSi_2_ ligand planes are tilted with respect to the EN_2_ plane in the crystal structure. In addition, they have close intramolecular contacts as well as different E–N–Si angles for the ‘inner’ (wider E–N–Si angle) and ‘outer’ (narrower E–N–Si angle) SiMe_3_ groups. The differences are *ca.* 12.3° for the Ge, *ca.* 11° for the Sn, and *ca.* 8.5° for the lead species. Inspection of the theoretical data show that there is good agreement with the calculated bond lengths for both the vapor and crystal phase structures. However, for the key N–E–N and E–N–Si angles, there is good agreement only in the case of the X-ray crystallographic data, where the calculated bond lengths, N–E–N angles, and the different ‘inner’ and ‘outer’ E–N–Si angles are faithfully reproduced in the calculations. The X-ray data show that the shortest E–E distances in the three crystal structures are 5.36 Å (Ge), 4.96 Å (Sn), and 6.663 Å (Pb). In the tin structure, the relative orientation of the tins is head to head between the closest monomers, and although there is a 0° torsion angle between the perpendiculars to the SnN_2_ planes, there is a displacement of the units with respect to each other such that imaginary lines bisecting the SnN_2_ angles are parallel to each other but are 0.52 Å apart.

**Table 4:** Calculated and experimental structural data for the E{N(SiMe_3_)_2_}_2_ (E = Ge, Sn, or Pb) monomers

Ge{N(SiMe_3_)_2_}_2_					
	B3PW91	B3PW91-D3	B97-D3	GED^*a*^	X-ray^*b*^
E–N (Å)	1.897 (avg.)	1.897	1.909	1.89(1)	1.875(3)
N–Si (Å)	1.778 (avg.)	1.765 (avg.)	1.780 (avg.)	1.743 (avg.)	1.752 (avg.)
N–E–N (°)	107.8	104.3 (avg.)	105.0	101(1.5)	107.1(4)
E–N–Si (°)	117.0 (avg.)	118.9 (avg.)	119.0 (avg.)	121.1 (avg.)	112.6(4)
					124.9(5)

Sn{N(SiMe_3_)_2_}_2_					
	B3PW91	B3PW91-D3	B97-D3	GED^*b*^	X-ray^*c*^
E–N (Å)	2.110	2.093	2.121	2.09 (avg.)	2.092(4)
					(2.096, 2.088)
N–Si (Å)	1.765 (avg.)	1.755 (avg.)	1.769 (avg.)	1.74	1.742
N–E–N (°)	106.4	102.0	102.7	91.2	104.7(2)
E–N–Si (°)	118.3 (avg.)	118.9 (avg.)	118.8 (avg.)	117.3	112.71(4)
					112.9(1)

Pb{N(SiMe_3_)_2_}_2_					
	B3PW91	B3PW91-D3	B97-D3	GED^*c*^	X-ray^*c*^
E–N (Å)	2.251	2.222	2.241	2.20(2)	2.24(2) (avg.)
					(2.260, 2.222)
N–Si (Å)	1.753	1.744 (avg.)	1.759 (avg.)	1.75 (avg.)	1.724 (avg.)
N–E–N (°)	106.5	101.3	101.8	91(2)	103.6(7)
E–N–Si (°)	117.9 (avg.)	118.5	118.5	119.6 (avg.)	112(1)
					120.2(5)

^*a*^ Ref. 7.

^*b*^ Ref. 4 and 5.

^*c*^ Ref. 5.

### Structural and thermodynamic data for the putative amido dimers [E{N(SiMe_3_)_2_}_2_]_2_

The calculated structural parameters and dissociation energies of the putative [E{N(SiMe_3_)_2_}_2_]_2_ dimers at various levels of optimization are given in Table 5. The calculated structural parameters for the E{N(SiMe_3_)_2_}_2_ units within the dimers are close to those calculated for the monomeric structures which match those experimentally determined by X-ray crystallography. The E–E distances undergo large contractions when dispersion force corrections are included. However, the shortest distances calculated for the germanium and tin amido dimers, even with the inclusion of such forces at the B3PW91-D3 level, are significantly longer (by *ca.* 1.4 and 0.7 Å) than the measured values and the values calculated at the same level for the alkyl dimers. The metal–metal distance calculated for the amide-ligated lead dimer (3.714 Å) by the B3PW91-D3 method is *ca.* 0.5 Å longer than the corresponding calculated distance (3.241 Å) in the dialkyl lead dimer. These distances indicate considerably weaker interactions between the group 14 elements for the amido derivatives. Thermodynamic data (Table 5) for the amido dimers also provide further evidence for weak association. The binding energies calculated without the inclusion of dispersion forces for the germanium, tin, and lead amido dimers at the B3PW91 level yield negative values of –5.0, –3.5, and –4.1 kcal mol^–1^, respectively, and show that the E–E interaction is disfavored (*cf.* positive values of 5.6, 12.0, and 0.1 for the alkyls, Table 1). In other words, monomeric structures are favored in each case. However, at the B3PW91-D3 level, which includes dispersion force effects, the energies change to 8.4, 19.8, and 15.6 kcal mol^–1^. Using the B97-D3 method affords similar but somewhat lower values of 7.9, 16.4, and 12.5 kcal mol^–1^. Single point MP2 calculations yield corresponding values of 5.1, 15.6, and 3.9 kcal mol^–1^. The overall picture that emerges from the calculations on the amides is that the dimerization tendency is present but weaker than it is in the alkyls, and that the driving force for the association of the monomers is due to dispersive attraction forces the –N(SiMe_3_)_2_ ligands. Further calculations on the thermodynamics of dissociation show that the Δ*G* energies for the process are –16.4, –18.0, and –10.8 kcal mol^–1^ at 25 °C and 1 atm, so that dissociation and monomeric structures are favored under these conditions. These findings are, of course, consistent with the structural and physical data. The much weaker E–E interactions in the amides in comparison with the alkyls is probably a result of electronic and steric factors. The more electronegative –N(SiMe_3_)_2_ ligand causes a larger HOMO–LUMO separation on the E atom, which would lower the extent of orbital interaction between the monomers. Also, the configurational differences between the –CH(SiMe_3_)_2_ and –N(SiMe_3_)_2_ (no *syn*, *syn*–*syn*, *anti* configurational change is possible for the amido ligands) may lead to greater steric hindrance, and hinder the association of the two monomeric fragments in the case of the amido ligand.

**Table 5:** Calculated structural and thermodynamic data (kcal mol^–1^) for the putative amido dimers [E{N(SiMe_3_)_2_}_2_]_2_

	[Ge{N(SiMe_3_)_2_}_2_]_2_			[Sn{N(SiMe_3_)_2_}_2_]_2_			[Pb{N(SiMe_3_)_2_}_2_]_2_		
	B3PW91	B3PW91-D3	B97-D3	B3PW91	B3PW91-D3	B97-D3	B3PW91	B3PW91-D3	B97-D3
E–E (Å)	5.841	3.798	3.902	4.548	3.514	3.589	11.242	3.714	3.771
E–N (Å)	1.899 (avg.)	1.886 (avg.)	1.915 (avg.)	2.119 (avg.)	2.113 (avg.)	2.136 (avg.)	2.260 (avg.)	2.241 (avg.)	2.252 (avg.)
N–E–N (°)	107.8 (avg.)	103.9 (avg.)	104.9 (avg.)	105.5 (avg.)	103.1 (avg.)	102.7 (avg.)	105.5 (avg.)	101.9 (avg.)	102.3 (avg.)

**[E{N(SiMe**_**3**_ **)**_**2**_ **}**_**2**_ **]**_**2**_ **→ 2 E{N(SiMe**_**3**_ **)**_**2**_ **}**_**2**_									
Δ*E*^*a*^	–5.0	8.4 (5.1)	7.9	–3.5	19.8 (15.6)	16.4	–4.1	15.6 (3.9)	12.5
Δ*E*^*b*^	–7.0	3.6		–7.4	11.7		–4.8	11.8	
Δ*H*^*c*^	–7.6	3.8		–8.0	11.6		–5.6	11.5	
–*T*Δ*S*^*c*^	–8.8	–16.0		–10.0	–15.3		–5.2	–15.1	
Δ*G*^*c*^	–16.4	–12.2		–18.0	–3.7		–10.8	–3.6	

^*a*^Dissociation energy (kcal mol^–1^). MP2 values are in parentheses.

^*b*^With ZPE and BSSE corrections.

^*c*^At 25 °C (298 K) and 1 atm.

### Persistent dimers of stannylenes: the case of [Sn{SiMe^*t*^Bu_2_}_2_]_2_

With these analyses in hand, it is informative to consider other stannylenes in the literature. Whilst a plethora of such species have been reported, the vast majority are monomeric in solution, and many with extremely sterically demanding ligands remain monomeric in the solid state.^14^ One notable exception is [Sn{SiMe^*t*^Bu_2_}_2_]_2_, described by Sekiguchi, Apeloig and co-workers in 2006.^52^ This species persists as a dimer in solution and, in contrast to the vast majority of literature examples, reacts as a dimer. It has a very short Sn–Sn distance of 2.6683(10) Å and essentially planar coordinated tin atoms, but the tin coordination planes subtend an angle of 44.62(7)° with respect to each other. This led the authors to propose that the Sn–Sn bonding was a result of a triplet–triplet interaction between the two stannylenes on the basis of calculations on the model species Sn_2_(SiMe_3_)_4_. While no full molecule calculations have been reported for [Sn{SiMe^*t*^Bu_2_}_2_]_2_ to investigate dispersion force contributions, a qualitative assessment can be made. One notable feature of dispersion force interactions is their dependence on length-scale and they are thought to be highly attenuated beyond the sum of the van der Waals radii of the interacting atoms.^53^ Thus, a qualitative assessment of dispersion forces can be undertaken by analysing for sub-van der Waals distances in structures.

**Fig. 3:**
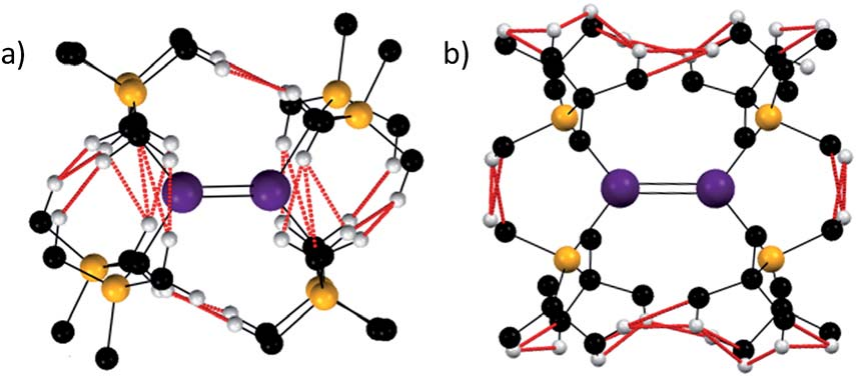
H–H and C–H distances below the sum of the van der Waals radii (2.4 and 2.9 Å respectively indicated in red) for [Sn{CH(SiMe_3_)_2_}_2_]_2_ (a) and [Sn{SiMe^*t*^Bu_2_}_2_]_2_ (b).

As shown, analysis of the crystallographically defined structures of [Sn{CH(SiMe_3_)_2_}_2_]_2_ and [Sn{SiMe^*t*^Bu_2_}_2_]_2_ is informative (Fig. 3). In the former case, two notable observations can be made- a number of monomer–monomer dispersion interactions are apparent, supporting the observed dimerisation in the solid state. Furthermore, whilst a *syn*, *anti* conformation is sterically disfavoured, a significant number of dispersion interactions can be observed between the ligands on each Sn{CH(SiMe_3_)_2_}_2_ unit indicating that such a sterically unfavourable conformation is partly stabilised by dispersion interactions.

In the case of [Sn{SiMe^*t*^Bu_2_}_2_]_2_, several dispersion interactions are evident between each of the interacting Sn{SiMe^*t*^Bu_2_}_2_ fragments, and they occur with greater frequency than in the [Sn{CH(SiMe_3_)_2_}_2_]_2_ species. Whilst the report^52^ of [Sn{SiMe^*t*^Bu_3_}_2_]_2_ provides an extensive electronic rationale for Sn–Sn interactions being responsible for its observed dimeric structure and short Sn–Sn distance, it is likely that dispersion forces are also of importance in stabilising the persistent dimeric nature of this compound.

To check this hypothesis, our initial full molecule calculations (see Table S5†) on [Sn{SiMe^*t*^Bu_2_}_2_]_2_ confirm the presence of a shorter tin–tin bond (2.647 Å) than that in [Sn{CH(SiMe_3_)_2_}_2_]_2_. However, the calculations show that the binding energy increases from 25.8 to 46.8 kcal mol^–1^ with inclusion of dispersion effects, and that the Δ*G* of dissociation at 25 °C and 1 atm is increased from 8.3 to a value of 26.5 kcal mol^–1^ upon inclusion of the dispersion correction, indicating that the dispersion force attraction is of key importance in maintaining the dimeric structure.

## Conclusions

The calculations have shown that the interplay between dispersion force attraction, steric repulsion and element–element bonding stabilize the dimeric structures. Although the E–E distances indicate E–E bonding is present in the germanium and tin dimers, and possibly the lead dimer, the bonding is weak, and represents a relatively small fraction of the binding energy. The results emphasize the importance of including attractive dispersion force interactions in consideration of multiple bonded heavier main group element species where sterically encumbering ligands are employed in their stabilization.^28^ Furthermore, these effects act in harmony with a variety of other stabilizing and destabilizing effects (*e.g.* packing or conformational) which are notable particularly for the –CH(SiMe_3_)_2_ ligand. A similar analysis for the non-dissociating distannene [Sn{SiMe^*t*^Bu_2_}_2_]_2_ shows that in this molecule also, the dispersion forces are of key importance in its stabilization. These studies provide an initial framework for the analysis of metal–metal bonding which takes into account these more subtle effects. Although much effort has been expended in development of bonding models for the multiple bonds between heavier main group elements, it seems both probable and ironic that the dispersion force attraction forces exceed those of the multiple bonds in many instances and that a variety of more subtle interactions including packing effects are of key importance for the understanding of their bonding.

## Supplementary Material

Supplementary information
