# Supplementary material for: The multiple bonding in heavier group 14 element alkene analogues is stabilized mainly by dispersion force effects
Source: Chem Sci. 2015 Aug 19;6(11):6235–44. doi: 10.1039/c5sc02707a (PMC6054042; doi:10.1039/c5sc02707a)
Supplement: Supplementary file 1 [file SC-006-C5SC02707A-s001.pdf]

Electronic Supplementary Material (ESI) for Chemical Science.
This journal is © The Royal Society of Chemistry 2015

The Multiple Bonding in Heavier Group 14 Element Alkene Analogues is Stabilized Mainly by Dispersion Force Effects

Jing-Dong Guo,[†] David J. Liptrot,[‡] Shigeru Nagase,*[,†] and Philip P. Power.*[,‡]

[†] *Fukui Institute for Fundamental Chemistry, Kyoto University, Takano-Nishiraki-cho 34-4, Sakyo-ku, Kyoto, Japan 606-8103, Japan.*

[‡] *Department of Chemistry, University of California, One Shields Avenue, Davis, California 95616, United States.*

Supporting Information

Contents

Table S1, Table S2, Table S3, Table S4	Page S2
Table S5	Page S3

Table S1. Calculated binding energies (kcal mol^{-1}) of E_2R_4 (E = Ge or Sn; R = $CH(SiMe_3)_2$) with a *syn*,*anti* configuration for the ER_2 units and H-C-E-C angles set at experimental values.

$[E\{CH(SiMe_3)_2\}_2]_2 \rightarrow 2E\{CH(SiMe_3)_2\}_2$				
	E = Ge		E = Sn	
	B3PW91	B3PW91-D3	B3PW91	B3PW91-D3
ΔE[a]	5.6 (-2.3)	40.2 (28.7)	12.0 (2.1)	38.5 (26.3)
ΔE[a,b]	14.0 (6.5)	50.4 (39.4)	13.3 (3.1)	40.5 (28.8)

[a] Binding energy. In parentheses are values with ZPE and BSSE corrections.
[b] The Ge and Sn monomers are optimized by fixing the (H-C-E-C) torsion angles at experimental values: 2° for GeR2; 15° for SnR2.

Table S2. Calculated structural data for the Ge_2R_4 and Sn_2R_4 (R = $CH(SiMe_3)_2$) dimers with the GeR_2 and SnR_2 units within the dimers in *syn*,*syn* configuration.

	Ge_2R_4			Sn_2R_4		
	B3PW91	B3PW91-D3	B97-D3	B3PW91	B3PW91-D3	B97-D3
E-E (Å)	2.428	2.377	2.452	2.842	2.765	2.855
E-C (Å)	2.034 (av.)	2.012 (av.)	2.051 (av.)	2.239 (av.)	2.210 (av.)	2.267 (av.)
C-E-C (°)	98.7 (av.)	98.1 (av.)	97.7 (av.)	96.3 (av.)	96.0 (av.)	95.3 (av.)
C-E-E (°)	122.6 (av.)	121.0 (av.)	120.2 (av.)	121.2 (av.)	119.2 (av.)	118.6 (av.)
C-E-E-C (°)	-51.6 (av.)<br>164.0 (av.)	-56.3 (av.)<br>167.2 (av.)	-58.7 (av.)<br>167.4 (av.)	-58.7 (av.)<br>168.7 (av.)	-62.9 (av.)<br>163.4 (av.)	-65.2 (av.)<br>166.9 (av.)
Trans-bent angle (°)	34.3 (av.)	38.0 (av.)	40.1 (av.)	39.0 (av.)	43.4 (av.)	44.8 (av.)

Table S3. Calculated binding energies (kcal mol^{-1}) of the E_2R_4 dimers (E = Ge or Sn; R = $CH(SiMe_3)_2$) with each ER_2 unit within the dimer in a *syn*,*syn* configuration.

$[E\{CH(SiMe_3)_2\}_2]_2 \rightarrow 2E\{CH(SiMe_3)_2\}_2$						
	E = Ge			E = Sn		
	B3PW91	B3PW91-D3	B97-D3	B3PW91	B3PW91-D3	B97-D3
ΔE[a]	-4.6	30.3 (29.6)	24.8	6.3	33.7 (34.0)	28.9
ΔE[b]	-14.1	18.9		-3.8	20.0	
ΔH[c]<br>-TΔS[c]<br>ΔG[c]	-13.5<br>-18.5<br>-32.0	20.0<br>-20.7<br>-0.7		-3.6<br>-16.6<br>-20.2	21.2<br>-21.5<br>-0.3	

[a] Binding energy. In parentheses are MP2 values.
[b] With ZPE and BSSE corrections.
[c] At 25℃ (298 K) and 1atm.

Table S4. Calculated and experimental structural parameters for the dimetallene Pb_2R_4 (R = $CH(SiMe_3)_2$), in which the $Pb\{CH(SiMe_3)_2\}_2$ units have the *syn*,*anti* configuration.

$[Pb\{CH(SiMe_3)_2\}_2]_2$				
	B3PW91	B3PW91-D3	B97-D3	X-ray[a]
E-E (Å)	3.202	2.956	3.073	4.129(1)
E-C (Å)	2.384(avg.)	2.345(avg.)	2.395(avg.)	2.313(5), 2.323(5)
C1-E-C2 (°)	106.3	106.6	106.4	93.4(2)
C-E-E (°)	108.7<br>121.7	108.3<br>116.7	107.4<br>119.4	

[a] Ref. 11.

Table S5. Calculations on the dissociation of $(SnR_2)_2 \rightarrow 2SnR_2$ (R = $SiMe^tBu_2$).

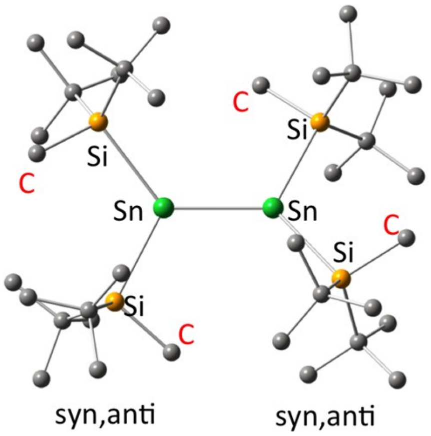

$(SnR_2)_2$

Unit: kcal/mol	Monomer: SnR_2 (R = $SiMe^tBu_2$)	
	B3PW91	B3PW91-D3
Singlet state	0.0	0.0
Triplet state	4.5	6.8
$(SnR_2)_2 \rightarrow 2\ SnR_2$ (R = $SiMe^tBu_2$)		
ΔE (dissociation)	36.4	59.5
ΔE (BSSE + ZPE)	25.8	46.8
(BSSE,25°C, 1.0 atm)		
ΔH	25.9	47.3
-TΔS	-17.6	-20.8
ΔG	8.3	26.5

	Dimer: $(SnR_2)_2$ (syn, anti)		
	B3PW91	B3PW91-D3	Exp.
Sn-Sn (Å)	2.702	2.647	2.668
Sn-Si (Å)	2.665	2.597	2.660 (av.)
Sn-Sn-Si (°)	124.6	124.3	113.8 (av.)
Si-Sn-Si (°)	110.9	111.4	111.7 (av.)
Trans-bending angle θ (°)	0.0	0.0	1.2
Torsion angle τ (°) (between Sn planes)	40.9	43.6	44.6
